# Lyme Disease, Anaplasmosis, and Babesiosis, Atlantic Canada

**DOI:** 10.3201/eid2806.220443

**Published:** 2022-06

**Authors:** Ziyad O. Allehebi, Farhan M. Khan, Mark Robbins, Elizabeth Simms, Richard Xiang, Allam Shawwa, L. Robbin Lindsay, Antonia Dibernardo, Clarice d’Entremont, Alex Crowell, Jason J. LeBlanc, David J. Haldane

**Affiliations:** Faculty of Medicine in Rabigh, King Abdulaziz University, Jeddah, Saudi Arabia (Z.O. Allehebi);; Dalhousie University, Halifax, Nova Scotia, Canada (Z.O. Allehebi, F.M. Khan, M. Robbins, E. Simms, J.J. LeBlanc, D.J. Haldane);; Nova Scotia Health, Halifax (Z.O. Allehebi, F.M. Khan, R. Xiang, A. Shawwa, J.J. LeBlanc, D.J. Haldane);; Public Health Agency of Canada National Microbiology Laboratory, Winnipeg, Manitoba, Canada (L.R. Lindsay, A. Dibernardo);; Yarmouth Regional Hospital, Yarmouth, Nova Scotia, Canada (C. d’Entremont, A. Crowell);; Nova Scotia Provincial Public Health Laboratory Network, Halifax (D.J. Haldane)

**Keywords:** babesiosis, babesia, Atlantic Canada, Nova Scotia, case, erythrocyte, ticks, *Ixodes scapularis*, *Borrelia burgdorferi*, tickborne diseases, vector-borne infections, bacteria

## Abstract

In July 2021, a PCR-confirmed case of locally acquired *Babesia microti* infection was reported in Atlantic Canada. Clinical features were consistent with babesiosis and resolved after treatment. In a region where Lyme disease and anaplasmosis are endemic, the occurrence of babesiosis emphasizes the need to enhance surveillance of tickborne infections.

Babesiosis is an emerging infectious disease caused by a zoonotic hemoprotozoan parasite of the genus *Babesia*, which consists of ≈100 species (*1*–*4*). Human disease in North America is primarily attributed to *Babesia microti*, and clinical features range from asymptomatic infection to severe disease or death (*1*–*6*). A small number of cases of locally acquired human *B. microti* infections in Central and Western Canada have been described (*3*–*6*). We report a confirmed case of babesiosis from Atlantic Canada in an area where Lyme disease and anaplasmosis are endemic (*7*–*9*). All clinical features and laboratory findings were consistent with babesiosis (*4*). 

A 58-year-old immunocompetent man sought care at a hospital in southwest Nova Scotia, Canada, in July 2021 for a 3-day history of nonspecific symptoms (headache, photophobia, fatigue, general weakness, and fever up to 40°C). The patient’s most recent travel was to Maine (USA) 25 years prior. He did not recall any recent tick bites; however, he had been treated for Lyme disease 3 times since 2019. 

At admission, laboratory results were unremarkable aside from elevated C-reactive protein (164 mg/L [reference range <8 mg/L]) and high lactate dehydrogenase (372 U/L [reference range 120–230 U/L]). Leukocyte counts were normal except for a new onset of thrombocytopenia (platelet count 73 × 10^9^/L). Wright-stained peripheral blood smears revealed intra-erythrocytic ring forms and extracellular merozoites (Figure). Parasitemia was estimated at 2.3%. Results of BinaxNow malaria testing was negative (Abbott Laboratories, https://www.globalpointofcare.abbott). *B. microti*–specific PCR performed on whole blood at the National Microbiology Laboratory (Winnipeg, Manitoba, Canada) was positive. 

**Figure:**
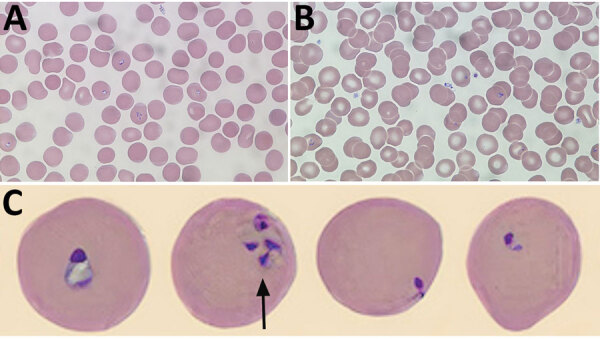
*Babesia microti* detected on Wright-stained peripheral blood smears from a 58-year-old man, southwest Nova Scotia, Canada, July 2021. Some typical features of *B. microti* infection include multiple ring forms present in erythrocytes (A), extracellular ring forms (B), and ring forms of various shapes and sizes (C), including the pathognomonic finding of merozoites arranged in a tetrad formation resembling a Maltese cross (arrow). Images in panels A and B obtained by using Wright’s stain (original magnification ×100), For panel C, the CellaVision DM96 system (https://www.cellavision.com) and the Cellavision Remote Review Software version 6.0.1 build 7 were used to capture and display cells with abnormalities.

On day 7 after symptom onset, the patient’s condition worsened, and parasitemia increased to 6.6%. Bloodwork showed increased C-reactive protein (298 mg/L), decreased platelets (56 × 10^9^/L), anemia (122 × 10^12^ erythrocytes/L), and increased liver enzymes (aspartate aminotransferase 76 IU/L [reference range 5–45 U/L], alanine aminotransferase 69 IU/L [reference range 0–54 U/L], and alkaline phosphatase 120 IU/L [reference range 38–150 U/L]). The patient was treated with atovaquone (750 mg orally 2×/d) and with azithromycin (500 mg orally 1×/d) for 10 days, along with doxycycline for 14 days for possible Lyme disease co-infection. Over the next 7 days, parasitemia gradually decreased to undetectable levels; the patient improved clinically and was discharged.

*B. microti* is primarily transmitted through feeding of infected nymphal and adult female ticks (*1*–*3*). In Atlantic Canada, the vector (*Ixodes scapularis* blacklegged ticks) and reservoir (the white-footed mouse *Peromyscus leucopus*) for *B. microti* are the same as those for *Borrelia burgdorferi* (*7*). Locally acquired *B. microti* infections are thought to be rare in Canada; previous cases were reported only recently from Central and Western Canada (*3*–*6*), and only rare occurrences are described in previous surveillance in human, animal, and ticks (*6*,*10*). Climate change and other environmental factors are now known to influence the abundance, range, and activity of ticks and reservoirs, as well as the risks for human exposure to tickborne pathogens (*1*,*10*). As seen with the increasing spread of *Ixodes ricinus* ticks in Europe (*1*), a northward expansion of blacklegged ticks is occurring in the southern parts of central and western Canada and in the Atlantic provinces, along with a concomitant rise in reported cases of Lyme disease (*10*). Compared with other provinces of Canada, Nova Scotia has the highest incidence of Lyme disease, increasing from 1.7 to 26.1 cases/100,000 population during 2009–2015 (*7*). Recently, increasing reports of ticks infected with *Anaplasma phagocytophilum* and cases of human granulocytic anaplasmosis also have been documented in Nova Scotia (*8*,*9*). This case of locally acquired *B. microti* infection adds another item to the menu of tickborne diseases in that Atlantic province. In absence of transovarial transmission in ticks with *B. microti*, expansion of the vector alone is unlikely to increase babesiosis cases unless sufficient amplification of the parasite is occurring in natural reservoirs. In northeast sections of North America, human infections caused by *B. microti* appear to be limited to the white-footed mouse, short-tailed shrew (*Blarina* spp.), and chipmunks (*Tamia striatus*) (*1*). Ongoing surveillance of tickborne disease in Atlantic Canada should include monitoring for *B. microti* in humans, ticks, and small mammals.

The discovery of *B. microti* infection in Atlantic Canada is important for multiple reasons. From a clinical perspective, physicians should be aware of the possibility of babesiosis occurring in the region, be able to recognize compatible symptoms, and be prepared to trigger proper investigations and implement therapeutic options when warranted. Because Lyme disease and anaplasmosis are already endemic in Nova Scotia, co-infections also should be considered if *B. microti* is detected; however, without evidence supporting the reciprocal conclusion, treatment of *B. microti* infection in cases of Lyme disease should only be considered if compatible with the clinical context (*8*,*9*). Ongoing surveillance, increased awareness, and education should be encouraged to better define and understand the changing epidemiology of tickborne diseases in Atlantic Canada.
