# Correction: Vol. 28, No. 6

**DOI:** 10.3201/eid3007.C23007

**Published:** 2024-07

**Keywords:** errata, erratum, correction

An affiliation for the first author was missing in Lyme Disease, Anaplasmosis, and Babesiosis, Atlantic Canada (Z.O. Allehebi et al.). The article has been corrected online (https://wwwnc.cdc.gov/eid/article/28/6/22-0443_article).

